# Changes to the European Resuscitation Council guidelines for adult resuscitation

**DOI:** 10.1016/j.bjae.2022.02.004

**Published:** 2022-04-20

**Authors:** A.D. Kane, J.P. Nolan

**Affiliations:** 1 James Cook University Hospital, South Tees NHS Foundation Trust, Middlesbrough, UK; 2 Warwick Clinical Trials Unit, University of Warwick, Coventry, UK; 3 Royal United Hospital, Bath, UK

**Keywords:** advanced life support, basic life support, cardiopulmonary resuscitation

**Learning objectives**

By reading this article you should be able to:

• Describe trends in the epidemiology of cardiac arrest.
• Be aware of changes in adult basic and advanced life support (ALS) guidelines.
• Appreciate that ALS may be modified in special circumstances.
• Be up to date in post-resuscitation care.

**Key points**

• About 8% of people having an out-of-hospital cardiac arrest survive to discharge.
• The community response (early recognition and access to emergency medical services, cardiopulmonary resuscitation and defibrillation) saves lives.
• Use a stepwise approach to airway management, moving on to more advanced techniques only if ventilation fails.
• Capnography is mandatory if tracheal intubation is performed. No trace = wrong place, even in cardiac arrest.
• The guidelines on neuroprognostication after cardiac arrest have been updated.

## The guideline process

Since 2000, cardiopulmonary resuscitation (CPR) guidelines have been produced on a 5-yearly cycle. Major updates have been published every 5 yrs interspersed with interim updates of specific topics as and when very important new science is published. The International Liaison Committee on Resuscitation (ILCOR) coordinates a continuous review of all resuscitation science and publishes its findings and broad treatment recommendations in an annual summary document.^1^ This organisation represents resuscitation organisations from most regions of the world and includes, for example, the European Resuscitation Council (ERC) and American Heart Association (AHA). Although ILCOR publishes systematic reviews of the highest quality, the relative lack of high-quality RCTs in resuscitation makes most of the evidence of low or very low certainty. Systematic reviews are supplemented by scoping reviews, which include broad search terms and lack bias assessments and meta-analyses. The lack of high-certainty evidence makes it difficult to make strong recommendations based on science alone and inevitably the treatment recommendations produced by ILCOR often lack the detail that would be required for clinical implementation. Thus, the regional resuscitation organisations, such as the ERC and the AHA, publish their own more detailed guidelines.^2^^,^^3^ In the absence of RCTs, many of these guidelines are based on expert consensus and are referred to as ‘good practice statements’. All ILCOR member organisations sign up to the principle that their clinical guidelines do not conflict with any recommendations published by ILCOR.

Although based on the 2020 ILCOR Consensus of CPR Science, because of the pressures on all healthcare systems caused by the COVID-19 pandemic, the publication of the ERC Guidelines was delayed until 2021. The guidelines published by the Resuscitation Council UK (RCUK) are adapted from those of the ERC and differ only in being more concise—this remained the case in 2021 (https://www.resus.org.uk/library/2021-resuscitation-guidelines).

The 2021 ERC Guidelines included the following topics:

• Epidemiology^4^
• Systems saving lives^5^
• Adult basic life support^6^
• Adult advanced life support^7^
• Special circumstances^8^
• Post-resuscitation care (in collaboration with the European Society of Intensive Care Medicine)^9^
• *First aid*
• *Neonatal life support*
• *Paediatric life support*
• *Ethics*
• *Education*

The purpose of this review is to highlight the main changes in the adult resuscitation guidelines; the topics listed in italics will not be covered here.

## Epidemiology

Recent publications derived from resuscitation registries have provided up-to-date data on incidence and outcome from both in-hospital cardiac arrest (IHCA) and out-of-hospital cardiac arrest (OHCA). The annual incidence of OHCA in Europe is between 67 and 170 per 100,000 population and resuscitation is attempted by emergency medical services (EMS) personnel in 50–60% of cases.^4^ In England, EMS personnel attempt resuscitation in 53 per 100,000 population annually.^10^ This represents 50% of all OHCAs.^11^ Rates of survival to hospital discharge after OHCA vary across Europe but on average are 8%, which is also the survival rate in England (https://warwick.ac.uk/fac/sci/med/research/ctu/trials/ohcao/map).^10^ In most European countries a favourable functional outcome is reported in >90% of hospital survivors, although more sophisticated testing reveals cognitive impairment in about half of these. However, in those few communities in which withdrawal of life-sustaining treatment (WLST) is not practiced, up to 50% of survivors may have a poor functional outcome.^4^

The annual incidence of IHCA in Europe is between 1.5 and 2.8 per 1000 hospital admissions. In the UK, most acute hospitals report IHCAs that include a 2222 call to the National Cardiac Arrest Audit (NCAA). The most recent NCAA summary statistics indicate a cardiac arrest rate of 1.0 per 1000 hospital admissions (https://www.icnarc.org/Our-Audit/Audits/Ncaa/Reports/Key-Statistics), but this will be an underestimate because cardiac arrests not accompanied by a 2222 call (e.g. many cardiac arrests in ICUs, emergency departments and cardiac catheterisation laboratories) are not included. This NCAA cardiac arrest rate has decreased considerably over the last 5 yrs—it was about 1.5 per 1000 hospital admissions in 2016. Survival rates have also increased during this time—the most recent data indicate a rate of survival to hospital discharge of 23.9%. The primary reason for the decrease in the incidence of cardiac arrest and increased survival rate is likely to be an increase in the use of do-not-attempt CPR decisions; however, there are no high-certainty data to prove this hypothesis.

## Systems saving lives

The early links in the chain of survival have a greater impact on survival from OHCA than the final link (post-resuscitation care). The community response is vital and efforts to improve recognition of cardiac arrest, immediate access to the EMS, early bystander CPR and use of public access defibrillation will all contribute to improving survival rates after OHCA.^5^ Bystander CPR increases survival rates by 2–3 times.^12^^,^^13^ Emergency dispatchers now routinely provide instructions to bystanders on how to perform chest compressions while awaiting the arrival of the EMS. Volunteer lay responders in the vicinity of the cardiac arrest can be instructed via text alerts to go to the scene and in some cases can first be directed to retrieve the nearest automated external defibrillator.^14^ Campaigns such as the World Restart a Heart Day (16 October each year) and Kids Save Lives are increasing substantially the number of people who are trained in CPR.^5^

## Adult basic life support

The 2021 ERC Guidelines for basic life support (BLS) are largely unchanged from those published in 2015; however, the ERC has also published guidelines for resuscitation of patients with confirmed or suspected COVID-19.^6^^,^^15^ It is widely accepted that mouth-to-mouth ventilation carries a substantial risk of transmission of SARS-CoV-2. Whether chest compressions alone generate aerosol, and therefore a substantial risk of transmission of SARS-CoV-2, remains uncertain. Until proved one way or the other, the ERC and the RCUK advise that precautions are taken to reduce the risk of disease transmission during the resuscitation of a person with known or suspected COVID-19. Lay persons are advised not to open the airway and not to place their face near to the person's mouth and nose. They should also consider placing a facemask or cloth/towel over the person's mouth and nose before performing chest compressions and public access defibrillation. Healthcare personnel are advised to don airborne precaution personal protective equipment (PPE) before starting chest compressions or making any airway interventions. Initial defibrillation attempts can be made using droplet precaution PPE (while other team members are donning airborne precaution PPE) because defibrillation is not thought to be an aerosol-generating procedure (although some animal data are suggesting otherwise).^16^ These guidelines are under continual review by the ERC and the RCUK and are likely to change as the effectiveness of vaccines alters the risk–benefit balance of these interventions.

## Adult advanced life support

**Fig 1:**
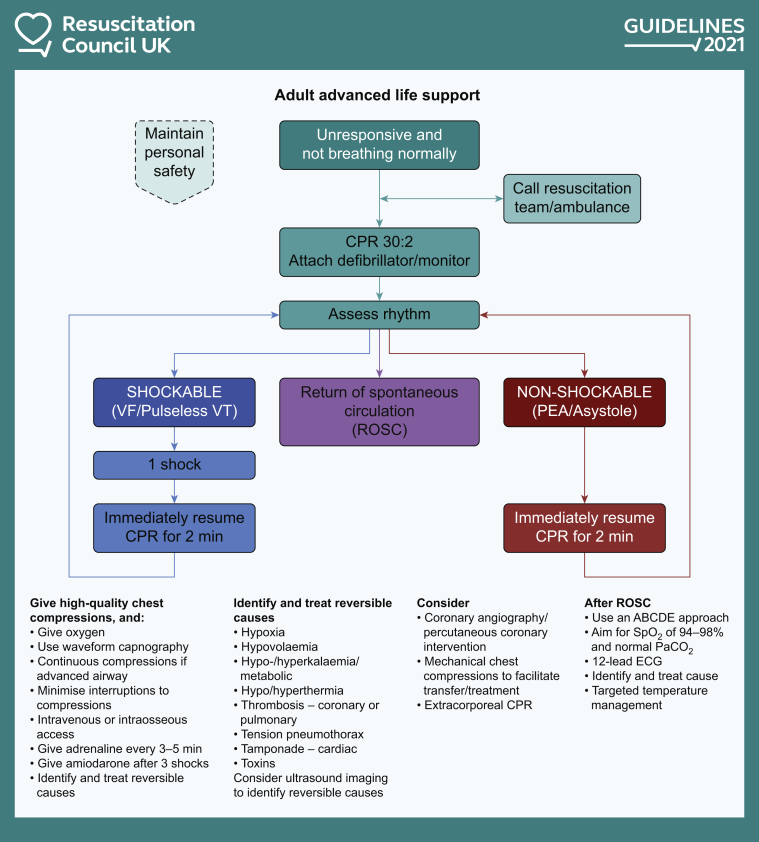
The 2021 Resuscitation Council UK adult advanced life support algorithm. Reproduced with the kind permission of Resuscitation Council UK. ABCDE, airway, breathing, circulation, disability, exposure/environment; EMS, emergency medical services; *P*aCO_2_, arterial partial pressure of carbon dioxide; PEA, pulseless electrical activity; SpO2, arterial oxygen saturation; VF, ventricular fibrillation; VT, ventricular tachycardia.

Although several important RCTs relating to advanced life support (ALS) have been completed since the last guidelines were published in 2015, these did not lead to major changes in the Adult ALS Guidelines (Fig. 1).^7^ Nevertheless, there have been some changes in emphasis in some interventions such as airway management, point-of-care ultrasound (POCUS) and use of extracorporeal CPR (eCPR).

### Airway management

Since 2015, two RCTs of airway management strategies in OHCA have indicated that the use of bag-mask ventilation or i-gel supraglottic airway device compared with an initial strategy of tracheal intubation results in at least equivalent outcomes.^11^^,^^17^ One RCT in the USA indicated that the use of the laryngeal tube by paramedics resulted in higher 72-h survival than the use of a tracheal tube; but the intubation success rate in this study was only 50%.^18^ Whether the results of these out-of-hospital studies can be generalised to IHCA is uncertain, but a time-dependent propensity analysis of a large IHCA registry in the USA showed that tracheal intubation within the first 15 min of cardiac arrest was associated with worse survival.^19^ A large RCT comparing tracheal intubation with supraglottic airway insertion in IHCA (AIRWAYS-3) is about to start in the UK. Having taken into consideration the results of these studies, the new ALS guidelines emphasise the importance of a stepwise approach to airway management in cardiac arrest until return of spontaneous circulation (ROSC) is achieved; this means starting with basic airway interventions (e.g. bag-mask ventilation) and progressing to more advanced techniques only if ventilation is ineffective.^7^ If an advanced airway is required, only rescuers with a high tracheal intubation success rate should attempt tracheal intubation. The expert consensus is that a high success rate is >95% within two attempts at intubation. Chest compressions should be interrupted for <5 s (or uninterrupted) during a tracheal intubation attempt. There are some data indicating that the use of videolaryngoscopy reduces the interruptions in chest compressions. The guidelines suggest using direct or video laryngoscopy for tracheal intubation according to local protocols and rescuer experience. Waveform capnography must be used to confirm tracheal tube position—carbon dioxide will be detected during CPR if the tracheal tube is in a major airway. If carbon dioxide is not detected, the tube is in the oesophagus (‘no trace = wrong place’) and must be removed.

### Drugs

The intraosseous (i.o.) route enables rapid delivery of drugs during cardiac arrest. In the UK, existing EMS protocols generally recommend that the i.o. route is attempted if two attempts at intravenous (i.v.) access have failed. The UK PARAMEDIC-3 study will randomise 15,000 patients with OHCA to either an i.o. first strategy or an i.v. first strategy (https://doi.org/10.1186/ISRCTN14223494) and may help to determine the precise role of the i.o. route in resuscitation.

### POCUS

The use of POCUS has increased in the emergency setting, as has its use during CPR.^20^ POCUS can be used to diagnose treatable causes of cardiac arrest including cardiac tamponade and pneumothorax, but the guidance emphasises that it should be performed by skilled operators. Interruptions in CPR must be minimised and a subxiphoid probe position during a planned rhythm check is advised. There are many limitations including overinterpretation of right ventricular (RV) dilation to indicate massive pulmonary embolism—RV dilation occurs in multiple aetiologies of cardiac arrest.^21^ An ILCOR systematic review also noted that no findings on POCUS had sufficient sensitivity to terminate CPR.^22^

### eCPR

Extracorporeal CPR is the establishment of veno-arterial extracorporeal membrane oxygenation in patients undergoing CPR who have failed to achieve ROSC.^23^^,^^24^ It aims to restore circulation and gas exchange and enable reversible causes of cardiac arrest to be addressed. A weak recommendation is made that eCPR can be considered when conventional CPR is failing in settings where it can be implemented. Setting up an eCPR service requires substantial resources^25^; the extent of its implementation internationally is highly variable.

## Special circumstances

The special circumstances section of the ERC guidelines outlines the modifications needed to BLS and ALS for cardiac arrest from specific special causes (e.g. hypoxia, trauma), settings (e.g. operating theatre, on transport [in-flight], mass casualty incidents), and patient groups (e.g. asthma, pregnancy), the full scope of which is beyond this article but some key highlights are summarised.^8^

Traumatic cardiac arrest (TCA) differs from medical cardiac arrest because it is typically caused by hypovolaemic, obstructive or neurogenic shock. In TCA focus is on the immediate simultaneous treatment of reversible causes including, where indicated: controlling external catastrophic haemorrhage, securing the airway and maximising oxygenation, bilateral chest decompression, relief of cardiac tamponade in penetrating chest injury, proximal vascular control (e.g. resuscitative endovascular balloon occlusion of the aorta/aortic compression), application of a pelvic binder and activation of a massive haemorrhage protocol. Where there is expertise, equipment, an appropriate environment and <15 min since the loss of vital signs, a resuscitative thoracotomy may be considered for tamponade or proximal vascular control, but should not be performed if these conditions are not met because it will expose the team to unnecessary risk and is likely to be futile.^8^

Whilst rare, of special note to anaesthetists is cardiac arrest in the operating theatre. The ALS algorithm should be followed while considering specific reversible causes which occur in this environment (e.g. hypovolaemia from haemorrhage, anaphylaxis and systemic local anaesthetic toxicity). There is a recommendation to use ultrasound to guide resuscitation and to consider open cardiac compression as an alternative to closed chest compressions. Where available, eCPR can be considered as a rescue therapy when conventional CPR is failing. There is likely to be much learning on this topic from the 7th National Audit Project of the Royal College of Anaesthetists, which is currently examining all cases of perioperative cardiac arrest in the UK over a 1 yr period.^26^

## Post-resuscitation care

**Fig 2:**
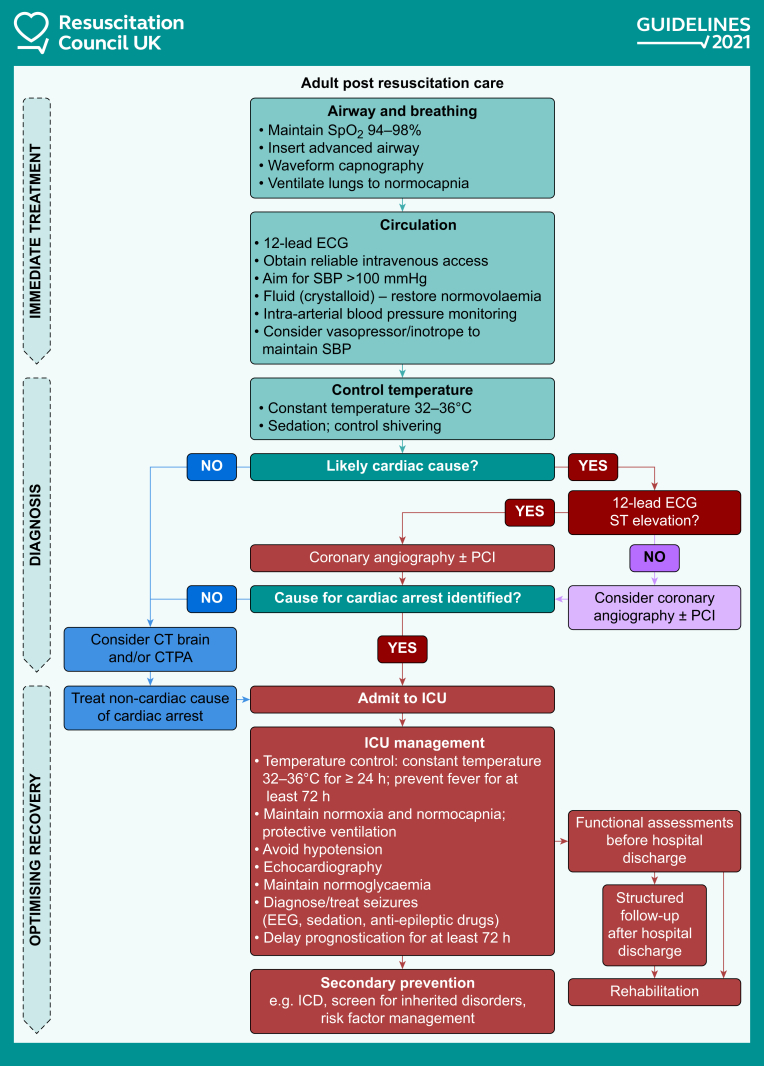
The 2021 Resuscitation Council UK post-resuscitation care algorithm. Reproduced with the kind permission of Resuscitation Council UK. CTPA, computed tomography pulmonary angiogram; ICD implanted cardioverter defibrillator; PCI, percutaneous coronary intervention; SBP, systolic BP; SpO_2_, arterial oxygen saturation.

After initial resuscitation and ROSC, the focus moves immediately towards post-resuscitation care, where there is an effort to determine the cause of the cardiac arrest and instigate specific treatment (e.g. cardiac catheterisation with or without PCI), alongside neuroprotective strategies (see Fig. 2). In time, this moves to prognostication and rehabilitation. There are several significant changes to post-resuscitation care in the 2021 guidelines.^9^

### Coronary angiography and percutaneous coronary intervention

Emergency cardiac catheterisation should be undertaken in patients with ROSC who have ST elevation on their ECG after OHCA. The absence of ST elevation does not exclude ongoing ischaemia, which may be suggested by ongoing haemodynamic or electrical instability, the patient's past medical history, warning symptoms preceding the cardiac arrest, or initial rhythm noted at cardiac arrest. An RCT comparing early *vs* late coronary angiography after ventricular fibrillation arrest without ST elevation, showed no benefit from early angiography, and another RCT published after the current guidelines were written also showed no benefit from early coronary angiography in all-rhythm OHCA.^27^^,^^28^ However, both of these studies excluded unstable patients, thus the guidance that emergency cardiac catheterisation be considered in unstable patients with ROSC after OHCA without ST elevation remains valid.

### Haemodynamic targets

Previous guidelines advising titration of BP to achieve a urine output of 1 ml kg^−1^ h^−1^ have been updated to suggest the avoidance of hypotension (MAP <65 mmHg) and achieving a urine output of 0.5 ml kg^−1^ h^−1^ with normal or decreasing lactate levels. Whilst it is not clear if augmenting MAP is of benefit, multiple observational studies show an association between hypotension and harm.^9^ Although the guidelines state a threshold value for BP, optimal MAP targets are likely patient specific. MAP is one of the main determinants of cerebral blood flow (CBF). There are relatively few data on ICP in post-arrest patients, but raised ICP is particularly likely to occur after asphyxial cardiac arrest. In many postcardiac arrest patients, CBF autoregulation is impaired or the lower limit is right-shifted. This means that hypotension may result in cerebral hypoperfusion and higher MAP values may be appropriate. An individualised approach including cerebral oxygen saturation or ICP monitoring may help to determine an optimal MAP, but use of such monitoring is not routine clinical practice.

### Neuroprotection

For neuroprotection, there is a significant update on temperature control and a minor update on the management of seizures. The Targeted Temperature Management after Cardiac Arrest Trial (TTM-trial), showed no difference in the primary outcome of all-cause mortality between a target of 33°C *vs* 36°C for 36 h.^29^ Following this trial, many units adopted a 36°C target. An RCT of comatose patients after cardiac arrest from non-shockable rhythms compared TTM at 33°C with normothermia and showed improved neurological outcomes at 90 days in the TTM 33°C group.^30^ Since the publication of the ERC 2021 Guidelines, an RCT comparing 33°C with active fever prevention among comatose survivors of OHCA showed no difference in mortality or neurological outcome.^31^ Having considered all this evidence, ILCOR has issued revised recommendations on temperature control after cardiac arrest and the ERC-European Society of Intensive Care Medicine guidelines have been updated to be consistent with these recommendations. In patients unresponsive after ROSC, fever (>37.7°C) should be actively prevented for at least 72 h.^32^ Temperature control can be achieved by exposing the patient, using antipyretic drugs, or if this is insufficient, by using a cooling device with a target temperature of 37.5°C.

Seizures increase cerebral metabolic demand and may potentiate neurological damage in the postcardiac arrest brain. To treat seizures, levetiracetam or sodium valproate are advised as first-line antiepileptic drugs, in addition to standard sedation. There is no evidence to support prophylactic treatment with antiepileptics, even though seizures occur in 20–30% of postcardiac arrest patients in intensive care. Phenytoin and fosphenytoin are no longer recommended as first-line antiepileptics after cardiac arrest, as they have negative inotropic and vasodilatory effects that increase episodes of hypotension.^33^

### Prognostication

**Fig 3:**
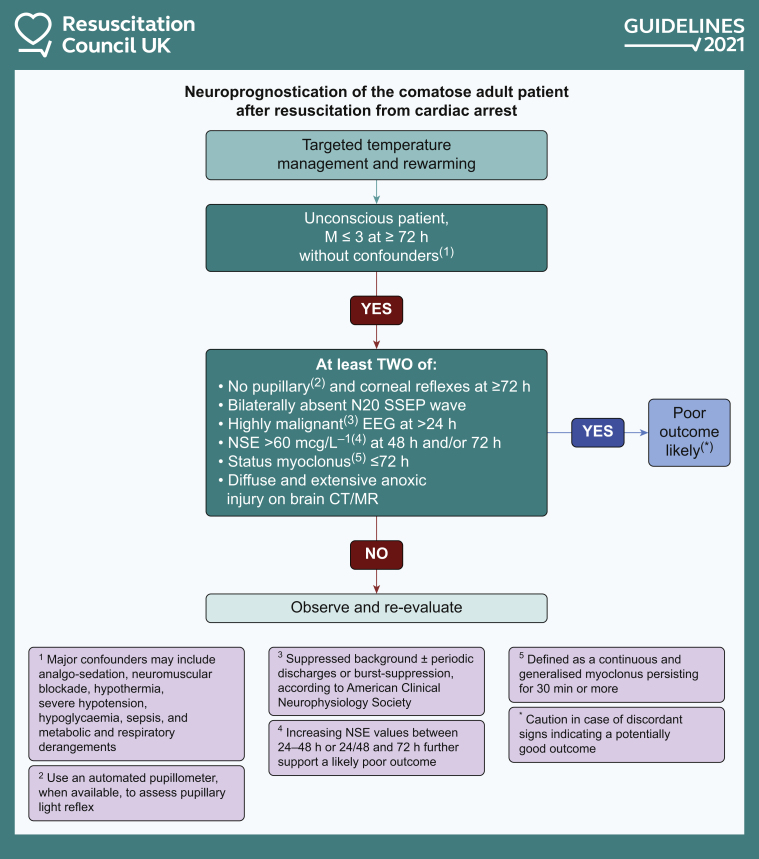
The 2021 Resuscitation Council UK prognostication algorithm. Reproduced with the kind permission of Resuscitation Council UK. MR magnetic resonance imaging, ; SSEP somatosensory evoked potential.

There have been significant advances in prognostication since the 2015 guidelines.^34^ These are reflected in the updated prognostication algorithm (Fig. 3).^9^ Prognostication should be delayed until at least 72 h, the patient should be rewarmed after TTM and confounders excluded (e.g. sedation, neuromuscular block, hypotension, hypoglycaemia, sepsis, metabolic or respiratory derangements). The patient should have a Glasgow motor score of ≤3. This has increased from ≤2 in the 2015 guidelines because it increases the sensitivity of predicting a poor outcome without reducing specificity. In these circumstances the presence of at least two of the following indicates a poor outcome is likely: no pupillary and corneal reflexes at ≥72 h, bilaterally absent N20 somatosensory evoked potential wave, highly malignant EEG (suppressed background or burst suppression) at >24 h, neuron-specific enolase (NSE) value >60 μg L^−1^ at 48 h, 72 h or both, status myoclonus ≤72 h, and, diffuse and extensive anoxic injury on brain CT or MRI. The NSE threshold value is new to the guideline—it should not be used alone to prognosticate. The likelihood of a poor neurological outcome is not synonymous with the WLST. There should be separate discussions about WLST that consider the patient's age, comorbidities and preferences, in addition to brain injury.

For those who survive there is increasing appreciation that rehabilitation and follow-up can improve quality of life. Functional assessment of physical and non-physical impairments before discharge will identify early rehabilitation needs. There should also be follow-up within 3 months to screen for cognitive and emotional problems and fatigue and to provide further opportunities for information giving and support to survivors and their families.

## Declaration of interests

JPN is editor-in-chief of the journal *Resuscitation*. AK declares no conflicts of interest.

## MCQs

The associated MCQs (to support CME/CPD activity) will be accessible at www.bjaed.org/cme/home by subscribers to *BJA Education*.
